# Systematic biases over the equatorial Indian Ocean and their influence on seasonal forecasts of the IOD

**DOI:** 10.1007/s00382-025-07794-6

**Published:** 2025-08-18

**Authors:** Marimel Gler, Andrew G. Turner, Linda C. Hirons, Caroline M. Wainwright, Charline Marzin

**Affiliations:** 1 Department of Meteorology, University of Reading, Reading, UK; 2 National Centre for Atmospheric Science, University of Reading, Reading, UK; 3 School of Earth and Environment, University of Leeds, Leeds, UK; 4 (formerly) School of Earth and Environmental Sciences, Cardiff University, Cardiff, UK; 5 Met Office, Exeter, UK

**Keywords:** Seasonal forecast, Mean state bias, Indian Ocean Dipole, IOD prediction

## Abstract

Accurate seasonal prediction of the Indian Ocean Dipole (IOD) is crucial given its socioeconomic impacts on countries surrounding the Indian Ocean. Using hindcasts from the Met Office Global Seasonal Forecasting System (GloSea6), coupled mean-state biases in the western and eastern equatorial Indian Ocean (WEIO and EEIO) and their impacts on IOD prediction are examined. Results show that GloSea6 exhibits a pronounced cold bias in the EEIO that rapidly develops after the monsoon onset in boreal summer (JJA, July–August) and persists into autumn (SON, September–November). This cold bias is linked to erroneous easterlies and a shallow thermocline, likely associated with the monsoon circulation. The seasonal evolution and relative timing of the precipitation biases, such that they develop through JJA in the EEIO but follow in the WEIO in SON, suggests that the EEIO plays the leading role in the development of coupled feedbacks that lead to the large dipole pattern of coupled biases. Analysis of skill metrics for the IOD shows that GloSea6 achieves a high anomaly correlation coefficient at short lead times, though it tends to overestimate IOD amplitude. This overestimation is larger in the eastern IOD pole than in the western pole and is likely linked to the poor representation of the evolution of the sea surface temperature anomalies in the EEIO during IOD events in SON. This study highlights the crucial role of regional biases, particularly in the EEIO, in shaping IOD variability and demonstrates that addressing such biases in GloSea6 could improve IOD prediction.

## Introduction

The tropical Indian Ocean and its interaction with the atmosphere modulate regional and global climate, and exhibit multiple modes of climate variability on intraseasonal-to-interannual timescales (Schott et al. 2009). The Indian Ocean Dipole (IOD) is the dominant coupled mode of interannual variability of sea surface temperature (SST) across the equatorial Indian Ocean (Saji et al. 1999; Webster et al. 1999). It is characterised by cool SST anomalies in the eastern equatorial Indian Ocean (EEIO) and warm anomalies in the western equatorial Indian Ocean (WEIO) during its positive phase, while the opposite pattern of SST anomalies occurs during its negative phase. Positive IOD events have been shown to increase flooding in East Africa (Wang and Cai 2020; Wainwright et al. 2021; Schwarzwald et al. 2023), and monsoon rainfall in India (Ashok et al. 2001; Hrudya et al. 2021) and Australia (Ashok et al. 2003; Saji and Yamagata 2003; Ashok et al. 2007; Cai et al. 2012; Liguori et al. 2022; Karrevula et al. 2024). A recent study by Karrevula et al. (2024) using the North American Multi-Model Ensemble seasonal forecasting system found that warming in the central Indian Ocean, driven by strong equatorial easterlies, plays a crucial role in modulating the frequency of extreme positive IOD events and their impact on summer monsoon precipitation from June to November.

The relationship between the IOD and the South Asian summer monsoon is complex and influenced by a range of coupled processes. While positive IOD events are often associated with enhanced monsoon rainfall over parts of India, the teleconnection is modulated by several factors including equatorial Indian Ocean dynamics, land–atmosphere interactions, and regional atmosphere circulation (Bollasina and Ming 2013; Annamalai et al. 2017; Crétat et al. 2017; Cherchi et al. 2021). Given the importance of regional climate and weather patterns influenced by the IOD, its accurate representation in models is crucial for producing reliable climate forecasts and future projections. Furthermore, since the IOD interacts with the El Ni$\tilde{\mathrm{n}}$o-Southern Oscillation (ENSO), accurately capturing the observed IOD characteristics is essential for improving forecasts of climate impacts on a global scale (Crétat et al. 2017; McKenna et al. 2020).    

Despite the socio-economic significance of the tropical Indian Ocean, the region suffers large mean state biases in general circulation models (GCMs) used for climate projections and seasonal forecasts (Li et al. 2015; Johnson et al. 2017; McKenna et al. 2020; Long et al. 2020; Marathe et al. 2021; Martin et al. 2021; Wang et al. 2021). Systematic biases during SON (September-November), when the IOD typically peaks and has significant regional climate impacts, have been found in the previous and latest generations of coupled GCMs that contribute to the Coupled Model Intercomparison Project (CMIP) (Li et al. 2015; Annamalai et al. 2017; Wang et al. 2021; Long et al. 2020). Earlier studies suggest that coupled biases over the equatorial Indian Ocean originate from spring and summer seasons, and are linked to biases in the simulation of the South Asian monsoon (e.g. Bollasina and Ming 2013; Prodhomme et al. 2014; Li et al. 2015; Annamalai et al. 2017). Li et al. (2015) found that these biases emerge during JJA, where a weakened South Asian monsoon leads to a warm SST bias over the western equatorial Indian Ocean, which is then amplified into SON via the Bjerknes feedback. On the other hand, Annamalai et al. (2017) found that the equatorial Indian Ocean bias originates earlier, in April–May, when easterly wind stress bias begins to develop across the equatorial Indian Ocean and persists through the JJA and SON seasons, peaking in November. This easterly wind stress bias from April–May initiates a warm SST bias in the western Indian Ocean that persists into JJA, ultimately influencing the summer monsoon. A more recent study by Long et al. (2020) demonstrated the source of the positive IOD-like pattern of the mean state biases in precipitation and SST across the equatorial Indian Ocean is linked to the warm SST bias in the western Indian Ocean, which is influenced by the South Asian summer monsoon circulation during JJA (June-August). This warm SST bias amplifies into SON via the positive Bjerknes feedback, a process driven by the zonal SST gradient across the equatorial Indian Ocean that strengthens low-level easterly winds and reinforces the west-east temperature gradient. The strong ocean–atmosphere coupling associated with the South Asian summer monsoon dominates the low-level circulation in the Indian Ocean during JJA, shaping the typical seasonal cycle of the IOD, which is observed to develop in JJA, peak in SON, and decay in boreal winter (DJF, December-February; Saji et al. 1999). Consequently, JJA and SON are key seasons for examining the predictability of the IOD and the development of coupled Indian Ocean biases. While the IOD typically develops during boreal summer and peaks in autumn, some events may begin earlier during boreal spring, with possible links to Indo-Pacific Ocean interactions. For example, Annamalai et al. (2003) suggest that equatorial Pacific SST anomalies can remotely initiate EEIO cooling and wind-driven upwelling off the coast of Sumatra, potentially triggering IOD events that are later sustained by local ocean–atmosphere feedbacks during JJA.

In a recent study, Mayer et al. (2024) showed that several current seasonal forecasting systems, provided by the Copernicus Climate Change Service (C3S 2018), share common mean state easterly wind and cold SST biases in the EEIO. For example, the fifth-generation European Centre for Medium-Range Weather Forecasts (ECMWF) seasonal forecast system (SEAS5) exhibits an easterly wind bias in the EEIO which develops within the first few days of the forecast and amplifies via coupled feedbacks, leading to a cold SST bias in the region (Mayer et al., 2022). On seasonal timescales, Mayer et al. (2024) attributed the cold bias to strong equatorial easterlies that induce a local easterly wind bias and shallow thermocline in the EEIO. This cold SST bias, arising from wind-induced upwelling, is further worsened by a shallow thermocline bias that already features in the EEIO oceanic initial conditions used.

Previous studies have shown that simulated mean state biases in the tropical Indian Ocean result in errors in the representation of the IOD (Zhao and Hendon 2009; Shi et al. 2012; Johnson et al. 2017; Hirons and Turner 2018; Wang et al. 2021). A mean state bias in the zonal SST gradient along the equatorial Indian Ocean, associated with a steep west-east upward tilt in the thermocline, leads to larger IOD amplitude compared to observations in climate and forecast models (Zhao and Hendon 2009; Wang et al. 2021). This is because a shallower thermocline in the mean state over the EEIO leads to local SSTs that are more susceptible to wind anomalies during IOD development, resulting in erroneous IOD SST anomalies (Johnson et al. 2017).

The development of such mean state biases in the equatorial Indian Ocean, along with poor initialisation of the subsurface ocean, have been shown to limit IOD predictability on seasonal timescales (Zhao and Hendon 2009; Liu et al. 2023). Liu et al. (2023) assessed the IOD predictability across two generations of seasonal forecast models, with the upgraded version demonstrating improved skillful prediction of the IOD of up to 6 months lead time, with a better simulated IOD spatial pattern and SST interannual variability, compared to its predecessor. The previous version exhibited a positive IOD-like bias in SST and zonal wind, resulting in stronger than observed cooling in the EEIO that extended too far west, accompanied by weak warming in the WEIO, during positive IOD events. They concluded that such a mean state bias in the tropical Indian Ocean led to an underestimation of the SST variability in the WEIO.

While some studies have focused on the sources of mean state biases in the equatorial Indian Ocean and others on the predictability of the IOD, very few have specifically linked these mean state biases to their impact on the prediction of the IOD. For example, although many of the aforementioned studies have highlighted persistent positive IOD-like biases in SST, circulation, and precipitation within coupled GCMs, most have not explored their effects on regional SST variability in the WEIO and EEIO, which are key poles of the IOD, and linked them to IOD prediction. Therefore, outstanding questions remain, that we aim to address in this study:

- How do mean-state biases in the atmosphere and subsurface ocean evolve in the WEIO and EEIO?
- What influence do the WEIO and EEIO regional biases have on the representation and predictability of the IOD?

In this study, we assess the performance of the UK Met Office Global Seasonal Forecasting System version 6 (GloSea6) in simulating the mean state and climate variability in the Indian Ocean, with a focus on the WEIO and EEIO regions. We examine the coupled ocean–atmosphere mean state biases and their interannual variability to better understand their influence on the representation of coupled dynamics and prediction skill of the IOD.

The remainder of this paper is structured as follows: a description of the forecast system, the observational data used, and the statistical methods applied is featured in Sect. 2. Section 3 contains the analysis of the development of mean state biases in SST, circulation and precipitation in JJA and SON, over the large-scale Indian Ocean, including the WEIO and EEIO. In Sect. 3, we further examine the coupled nature of the biases, by investigating the subsurface ocean compared to observations, evaluate the representation of the IOD spatial pattern and SST variability, and examine the prediction skill of SST anomalies associated with the IOD. Section 4 summarises the results and concludes the paper.

## Data and methods

### Model description

GloSea6 is an ensemble prediction system that is fully coupled with atmosphere, land surface, ocean, and sea-ice components. GloSea6 in Global Configuration 3.2 (GC3.2) consists of the following components: the Met Office Unified Model (UM) Global Atmosphere version 7.2, the Nucleus for European Modeling of the Ocean Global Ocean version 6.0, the Joint U.K. Land Environment Simulator Global Land version 8.0, and the Los Alamos Sea Ice Model Global Sea ice version 8.1. The atmosphere and land models are based on Walters et al. (2019), and the ocean and sea ice models are based on Storkey et al. (2018) and Ridley et al. (2018), respectively. The atmospheric model resolution is N216, corresponding to horizontal grid spacings of approximately 70 km in the tropics, with 85 vertical model levels extending up to 85 km. The ocean model has a horizontal resolution of 25 km, equivalent to 0.25° (ORCA025), with 75 vertical levels. MacLachlan et al. (2015) provide detailed model information on GloSea5, an earlier version of GloSea6 with the same atmospheric horizontal resolution. Both versions of GloSea produce sub-seasonal to seasonal forecasts for operational use, alongside corresponding hindcasts, and employ the same Stochastic Kinetic Energy Backscatter (SKEB) scheme to generate perturbations between ensemble members initialised from the same analysis (Bowler et al. 2009). The SKEB scheme introduces small, random perturbations to the wind field during model integration to represent uncertainty from unresolved sub-grid processes, re-injecting a portion of the kinetic energy lost through the semi-Lagrangian advection scheme, thereby increasing ensemble spread and improving the representation of forecast uncertainty.

In this study, monthly operational hindcasts are analysed to examine the Indian Ocean climate variability, and predictability of the IOD. GloSea6 uses a lagged initialisation approach to represent uncertainties in the initial conditions, with hindcasts initialised on the 1st, 9th, 17th, and 25th of every month from 1993 to 2016. Within the GloSea6 system, each start date has seven ensemble members, resulting in a total of 28 members each month. Ensemble members initialised on the 1st of the month are integrated longer for seven complete calendar months, including the month of initialisation, while those initialised on the 9th, 17th and 25th produces forecasts for six complete months.

Lead time in this study is defined as the number of calendar months elapsed since forecast initialisation. Forecasts at 0-month lead time (LM0) refer to the first complete calendar month of forecast output. Therefore, for GloSea6 hindcasts initialised on the 1st of the month, LM0 corresponds to that same calendar month, as the forecast begins on day one and spans the entire month. In contrast, for hindcasts initialised later in the month (on the 9th, 17th, or 25th), LM0 corresponds to the following calendar month, as GloSea6 outputs forecasts as monthly means starting from the first completed calendar month after initialisation. For example, LM0 for a 1st February start date corresponds to February, while LM0 for 9th, 17th, and 25th February start dates corresponds to March. Accordingly, monthly climatologies are constructed by averaging forecasts for the same calendar month across all relevant start dates. For instance, the March SST climatology at LM0 includes March forecasts initialised on 9th, 17th, and 25th February, and 1st March, averaged over all years from 1993 to 2016.

To assess the seasonal mean by lead time, monthly hindcasts with the same lead time are averaged to produce a hindcast seasonal mean. For example, the JJA mean at LM0 is created by averaging the first month of forecasts for June, July, and August. Likewise, the SON mean at a 0-month lead time is an average of the forecasts for September, October, and November, with each forecast started at the beginning of each month. By using this method, the influence of model drift is expressed equally in all three months.

#### Observational datasets

The fifth-generation ECMWF reanalysis (ERA5; Hersbach et al. 2020) at horizontal resolution 0.25° × 0.25°, is used for comparison with model output for dynamic fields such as 10 m and 850 hPa winds. For precipitation fields, the Global Precipitation Climatology Project (GPCP) dataset at 2.5° × 2.5° horizontal resolution, with monthly version 2.3 (Adler et al. 2003) and the Tropical Rainfall Measuring Mission (TRMM) Multi-satellite Precipitation Analysis monthly product, 3B43, constructed by the National Aeronautics and Space Administration at 0.25° × 0.25° horizontal resolution are used.

For verification with GloSea6 SST outputs, monthly SST from the Met Office Hadley Centre Sea Ice and Sea Surface Temperature (HadISST) dataset (Rayner et al. 2003) and National Oceanic and Atmospheric Administration Optimum Interpolation Sea Surface temperature version 2 (OISSTv2) monthly data are used (Reynolds et al. 2007). The ECMWF Ocean Reanalysis System 5 (ORAS5) is used for comparison against the GloSea6 ocean potential temperature in the subsurface (Zuo et al. 2019).

### Methods

The pattern correlation coefficient (PCC) and root mean square error (RMSE) are calculated with respect to observations to quantify the performance of GloSea6 in simulating the Indian Ocean mean climate and variability. PCC measures the degree of similarity between the spatial patterns of the observed and simulated fields, while RMSE measures the magnitude of the difference in simulation relative to observations. To assess the statistical significance of the difference between the simulated and observed Indian Ocean mean states, the paired Student’s t-test (Wilks, 2011) is performed on the hindcast ensemble mean and observations.

Observed and predicted IOD events are identified using the Dipole Mode Index (DMI), which is defined by the west-east gradient of SST anomalies between the western equatorial Indian Ocean (WEIO; 50–70°E, 10°S-10°N) and eastern equatorial Indian Ocean (EEIO; 90–110°E, 10°S-0°) (Saji et al. 1999). SST anomalies of the DMI timeseries are calculated relative to the full validation hindcast period of 1993–2016. To quantify the performance of GloSea6 in predicting the IOD, deterministic metrics such as the anomaly correlation coefficient (ACC) and root-mean-square error (RMSE) are evaluated. These metrics are calculated between the observed and predicted SST anomaly time series of the DMI. To compare the IOD variability between GloSea6 and observations, the amplitude ratio is computed, defined as the ratio of the standard deviation of the predicted DMI to that of the observed DMI (e.g. Johnson et al. 2019; Wedd et al. 2022). An amplitude ratio < 1 indicates that the model underestimates IOD variability compared to observations, while a ratio >1 suggests that the model overestimates it.

## Results

In this section, the ability of GloSea6 to capture the observed climatological JJA and SON mean states, in the atmosphere and subsurface ocean, is assessed. Given the importance of JJA and SON on the seasonality of the development and maturity of the IOD, respectively, we evaluate the simulated seasonal evolution of coupled processes with respect to observations. Specifically, we examine the biases related to monsoon circulation in JJA that influence the coupled ocean–atmosphere Bjerknes feedback across the equatorial Indian Ocean in SON.

### Development of coupled ocean–atmosphere biases in JJA and SON

**Fig. 1:**
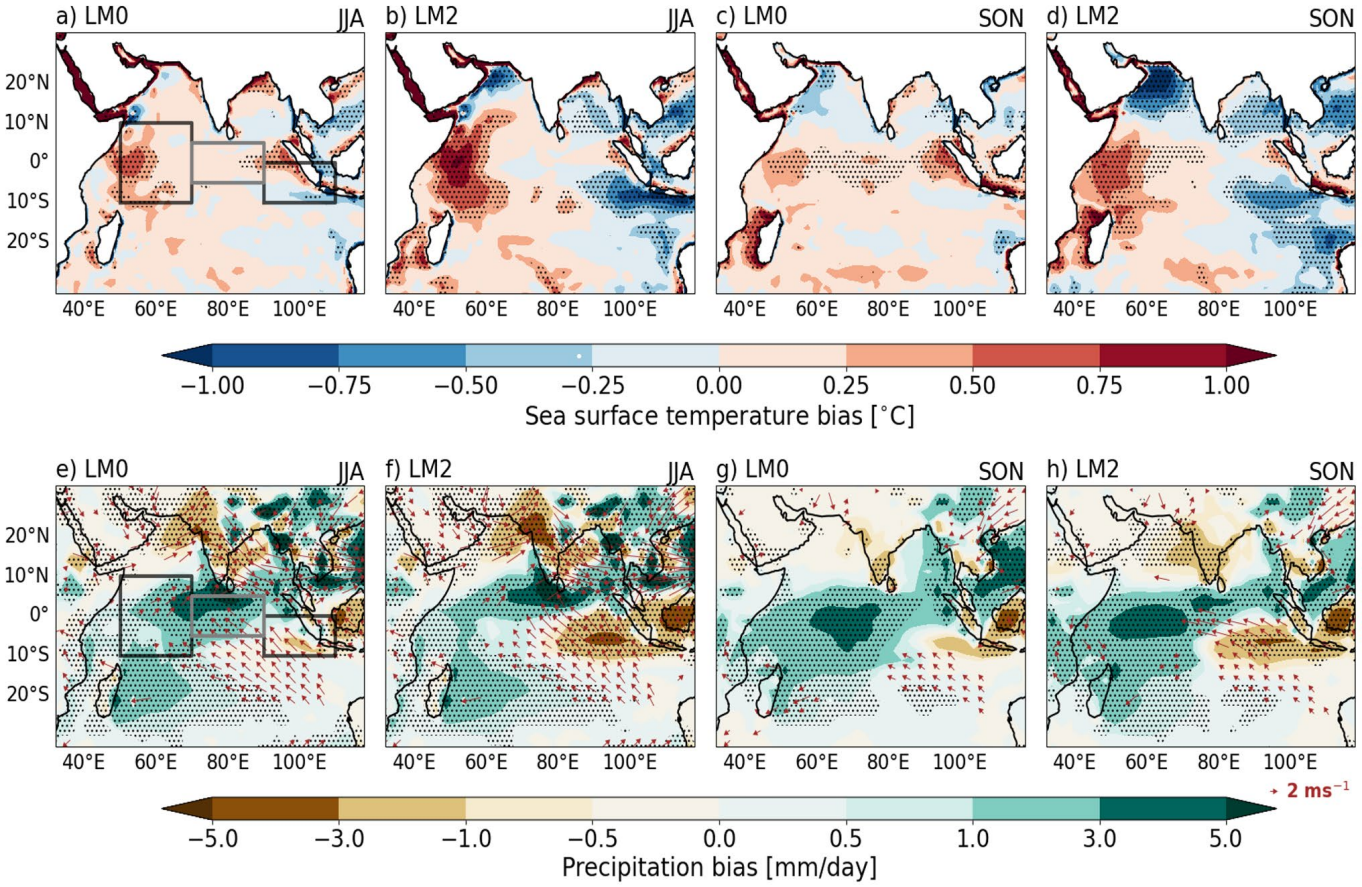
Climatological JJA and SON mean biases in GloSea6 for (**a**–**d**) SST, and (**e**–**h**) precipitation and 850 hPa winds at 0-month (1st month of the forecast; LM0) and 2-months lead time (3rd month of the forecast; LM2). GloSea6 SST, precipitation and low-level winds are compared against HadISST, GPCP and ERA5, respectively, from 1993–2016. Black boxes show the western (50–70°E, 10°S-10°N) and eastern (90–110°E, 10°S to equator) poles of the IOD. Grey box shows the central equatorial Indian Ocean (70–90°E, 5°S-5°N), used to capture a metric of zonal wind. Black stipples on the SST and precipitation panels indicate regions where these mean-state biases are statistically significant at the 95\% confidence level, based on a paired Student’s t-test. The overlaid 850 hPa wind vectors are shown only where they are also significant at the same confidence level

Figure 1 compares the JJA and SON mean state biases in SST, precipitation, and 850 hPa winds at LM0 (0-month lead time) and LM2 (2-month lead time), showing how these biases differ between seasons and how they change with increasing lead time. Across the equatorial Indian Ocean, GloSea6 exhibits a predominantly warm SST bias, with a small but significant cold bias over the EEIO during JJA at LM0 (Fig. 1a). As lead time increases to LM2, this JJA SST bias intensifies into a distinct and significant dipole pattern, characterised by a warm SST bias in the WEIO and a cold SST bias in the EEIO (Fig. 1b). The SON SST bias follows a similar evolution: starting with a significant warm bias across much of the tropical Indian Ocean, which is largest over the EEIO at LM0 (Fig. 1c). By LM2, this bias develops into a dipole pattern resembling that of JJA at LM2, with pronounced warming in the WEIO and cooling in the EEIO (Fig. 1d). Although the evolution of SST bias into a dipole pattern is similar for JJA and SON with increasing lead time, the magnitude of the warming in the WEIO and cooling in the EEIO at LM2 is notably larger in JJA compared to SON. At LM4 and LM6, the dipole structure of the JJA and SON SST biases becomes well established across the equatorial Indian Ocean (not shown).

The dipole pattern of JJA and SON SST biases at LM2 resembles the SST anomalies typically observed during a positive IOD event (Saji et al. 1999). Previous studies (e.g., Johnson et al. 2017; Martin et al. 2021; Mayer et al. 2024) found a similar positive IOD-like pattern of JJA mean SST bias in GloSea5 and SEAS5 hindcasts. In GloSea6, the JJA and SON biases in precipitation and lower-tropospheric circulation (Fig. 1e–h) are consistent with the changes in SST biases as lead time increases. A dry bias over India in JJA (a known problem in the GloSea forecast model; Johnson et al. 2017; Martin et al. 2021; Keane et al. 2024) worsens from LM0 to LM2, while a dipole between excessive rainfall in the central Indian Ocean and a dry bias in the EEIO, off the coast of Sumatra, increases (Fig. 1e and f). Similarly, the SON biases in precipitation and circulation over the equatorial Indian Ocean show comparable changes, with significantly strengthened southeasterlies and a dry bias in the EEIO, alongside a wet bias in the WEIO by LM2 (Fig. 1e–h). However, it is notable that the SON precipitation bias is larger in the WEIO at LM2, despite responding to a smaller magnitude of SST bias, compared to the JJA precipitation bias at the same lead time. This may be related to the significantly stronger easterlies in the central equatorial Indian Ocean in SON compared to JJA at LM2, which likely enhances low-level convergence in the WEIO (Fig. 1f and h). A positive IOD-like precipitation pattern, with a wet western and central equatorial Indian Ocean and a dry EEIO, is established at LM2 in JJA and SON. These features are likely associated with the Bjerknes coupled feedback, where excessive easterly winds in the equatorial Indian Ocean are coupled with biased dipole patterns in SST and precipitation. For instance, the significant erroneous southeasterly flow off the coast of Sumatra enhances upwelling, which cools the SST further in that region, reinforcing the dipole pattern. The interactions between SST, winds, and precipitation leads to a coupled feedback loop that amplifies the initial biases and their associated patterns.

To investigate the interplay between SSTs, precipitation, and the subsurface ocean, and to further examine how ocean–atmosphere biases evolve from months to seasons ahead in the tropical Indian Ocean, quantities were averaged over the WEIO and EEIO regions. Analysis was performed on hindcast ensemble means initialised between February and November.

**Fig. 2:**
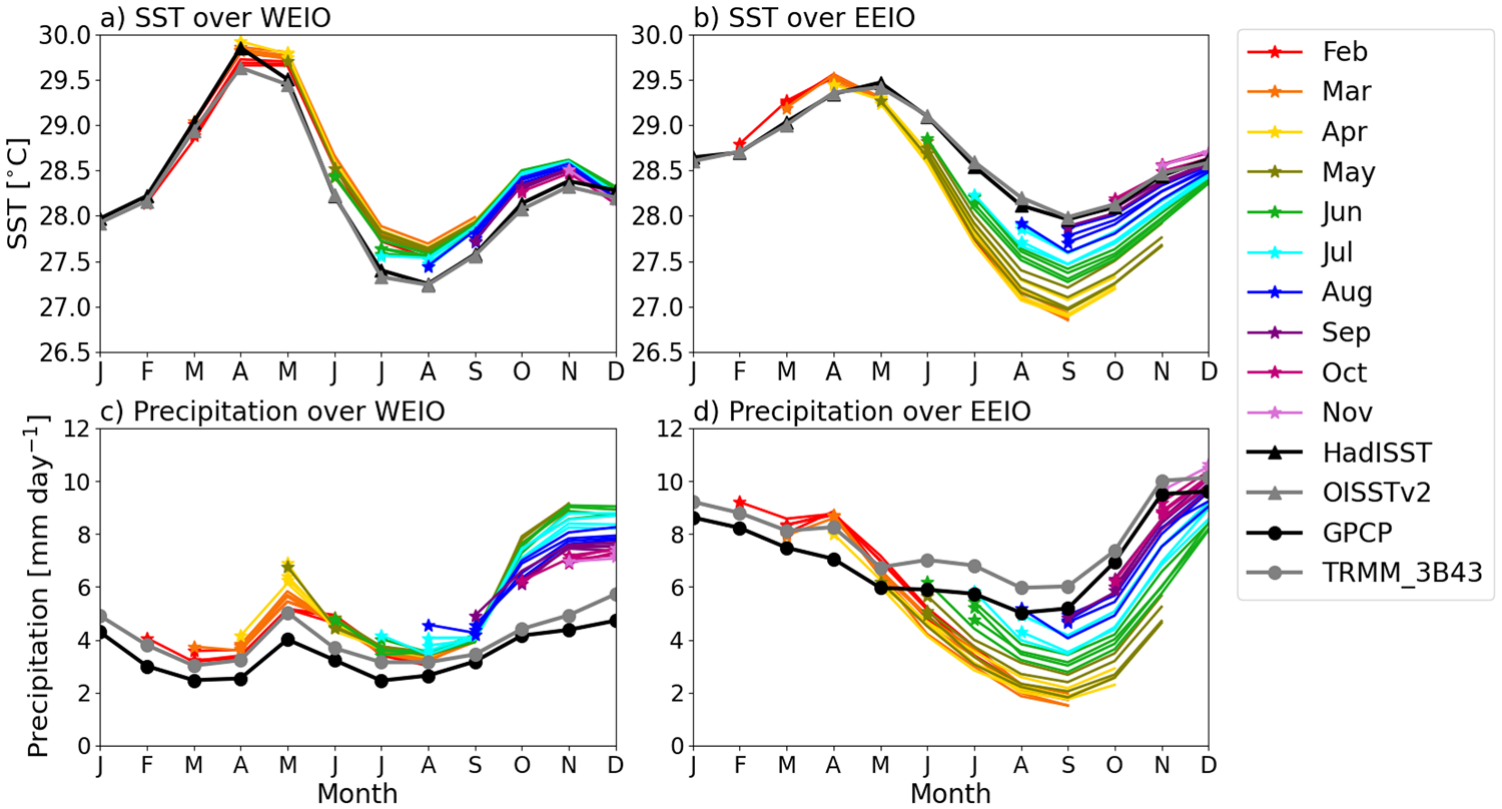
Monthly evolution of climatological (**a** and **b**) SST (against HadISST and OISSTv2) and **c** and **d** precipitation (against GPCP and TRMM_3B43) over the WEIO and EEIO in GloSea6 hindcasts initialised from February to November over the 1993–2016 hindcast period. Solid coloured lines represent the monthly ensemble means from 28 members: seven from each of four monthly start dates (1st, 9th, 17th, 25th)

Figure 2 shows the predicted climatological seasonal cycles of SST and precipitation compared to observations over the WEIO and EEIO. The SST in the WEIO (Fig. 2a) generally tends to be initialised systematically warmer than observations from May onwards in contrast to the EEIO (Fig. 2b). The EEIO SST bias initially shows warming for February–April start dates, but then rapidly develops into a cold bias from May onwards, persisting through JJA during the boreal summer monsoon and into SON when initialised from May-August starts. The distinct EEIO cold bias is much larger in magnitude than the warm bias in the WEIO, and is notably larger when initialised from February to July compared to the relatively smaller cold bias that develops following August and September initialisations. Forecasts running through a larger portion of the JJAS season tend to suffer a worse bias. Together with the circulation bias seen off Sumatra in Fig. 1e–h, this finding suggests that the northern hemisphere monsoon in JJA strongly influences the evolution of the SST bias in the EEIO. This indicates a strong seasonal dependence in the development of the EEIO SST bias. As in the case of the EEIO cold SST bias, hindcasts started from May–August show rapid growth of dry bias into the SON months (Fig. 2d), showing a strong seasonal dependence. The dry bias for hindcasts initialised in the autumn is much smaller, after the withdrawal of the boreal summer monsoon. Meanwhile in the WEIO (Fig. 2c), large precipitation biases do not begin to develop until autumn, coinciding with the positive IOD-like precipitation pattern of wet bias in the WEIO and dry bias in the EEIO during SON, which is also consistent with Fig. 1h. The more pronounced SST bias in the EEIO and the relative timing of the precipitation biases between the EEIO and WEIO, such that the biases develop through summer in the EEIO but only begin in the autumn in the WEIO, suggest that the EEIO plays a leading role in the development of the overall SST bias pattern. We note that the observational uncertainty in precipitation is generally larger compared to SST due to the highly variable nature of precipitation, which may contribute to some of the discrepancies seen in these biases. For instance, GPCP and TRMM_3B43 show a discrepancy of approximately 0.5–1 mm/day from January-September in the WEIO and EEIO (Fig. 2c) in contrast to the small and negligible monthly differences between HadISST and OISSTv2 throughout the year.

**Fig. 3:**
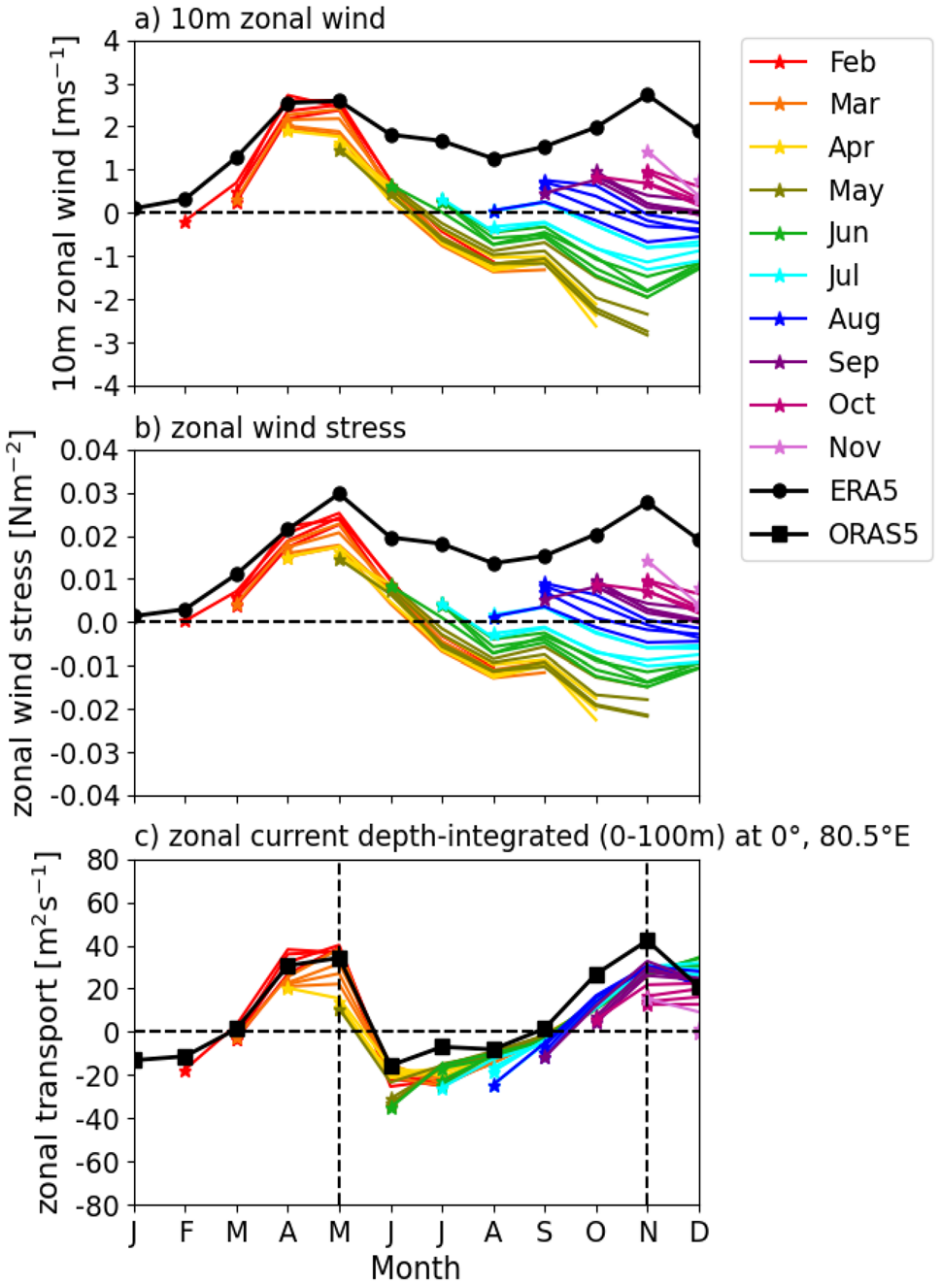
Monthly evolution of climatological **a** 10 m zonal wind (against ERA5), **b** zonal wind stress (against ERA5) over the central equatorial Indian Ocean (70–90°E, 5°S-5°N), as marked in Figure 1, which depicts the region used for capturing the metric for zonal winds, and **c** the Wrytki jet, measured as the depth-integrated (0–100 m) of zonal current (against ORAS5) at 0°, 85°E, adapted from Annamalai et al. (2017), in GloSea6 hindcasts initialised from February to November over the 1993–2016 hindcast period. Solid coloured lines represent the monthly ensemble means from 28 members: seven from each of four monthly start dates (1st, 9th, 17th, 25th). Dashed vertical lines during May and November illustrate the time when the Wyrtki jet peaks in ORAS5

In the central equatorial Indian Ocean, easterly wind biases in near-surface 10 m zonal winds and zonal wind stress develop in late spring, then rapidly intensify through JJA, and peak in SON (Fig. 3a and b). In particular, GloSea6 exhibits a weak easterly wind stress bias in March-April when initialised in February and March. As a result, the eastward-flowing Wyrtki jets remain relatively well developed in March and April for these early initialisations, compared to ERA5 (Fig. 3c). These jets are strong equatorial ocean currents that transport mass and heat in the upper ocean from the western to the eastern Indian Ocean biannually, during the spring and autumn intermonsoon seasons, driven primarily by westerly winds (Schott and McCreary, 2001). Therefore, the opposing easterly wind stress bias acts to suppress these eastward-flowing Wyrtki Jets, which is particularly evident for hindcasts initialised in April and May. The easterly wind bias is especially strong in May, resulting in considerably weaker Wyrtki jets relative to ORAS5. Notably, despite differences in the magnitude of the easterly wind stress bias in April–May across different initialisation months, this bias rapidly intensifies from May to June following the onset of the summer monsoon, and continues to strengthen through JJA and into SON.

The timing of the evolution of the biases in the equatorial Indian Ocean therefore appears to follow the sequence of substantial EEIO SST and precipitation biases from May (Fig. 2b and d). This is followed by the rapid growth of the erroneous zonal SST gradient, characterised by a larger cold bias in the east than the smaller warm bias in the west (Fig. 2a and b), and the central equatorial Indian Ocean wind biases in JJA (Fig. 3a and b), and then the WEIO precipitation biases in SON (Fig. 2c). This structure of the coupled biases indicates that they arise from Bjerknes feedback in the equatorial Indian Ocean, emerging from the atmospheric bias in the EEIO driving substantial SST and thermocline depth biases in the region, which in turn increases the zonal SST gradient across the equatorial Indian Ocean and strengthens the easterlies in the central equatorial IO, which leads to large precipitation bias in the WEIO.

**Fig. 4:**
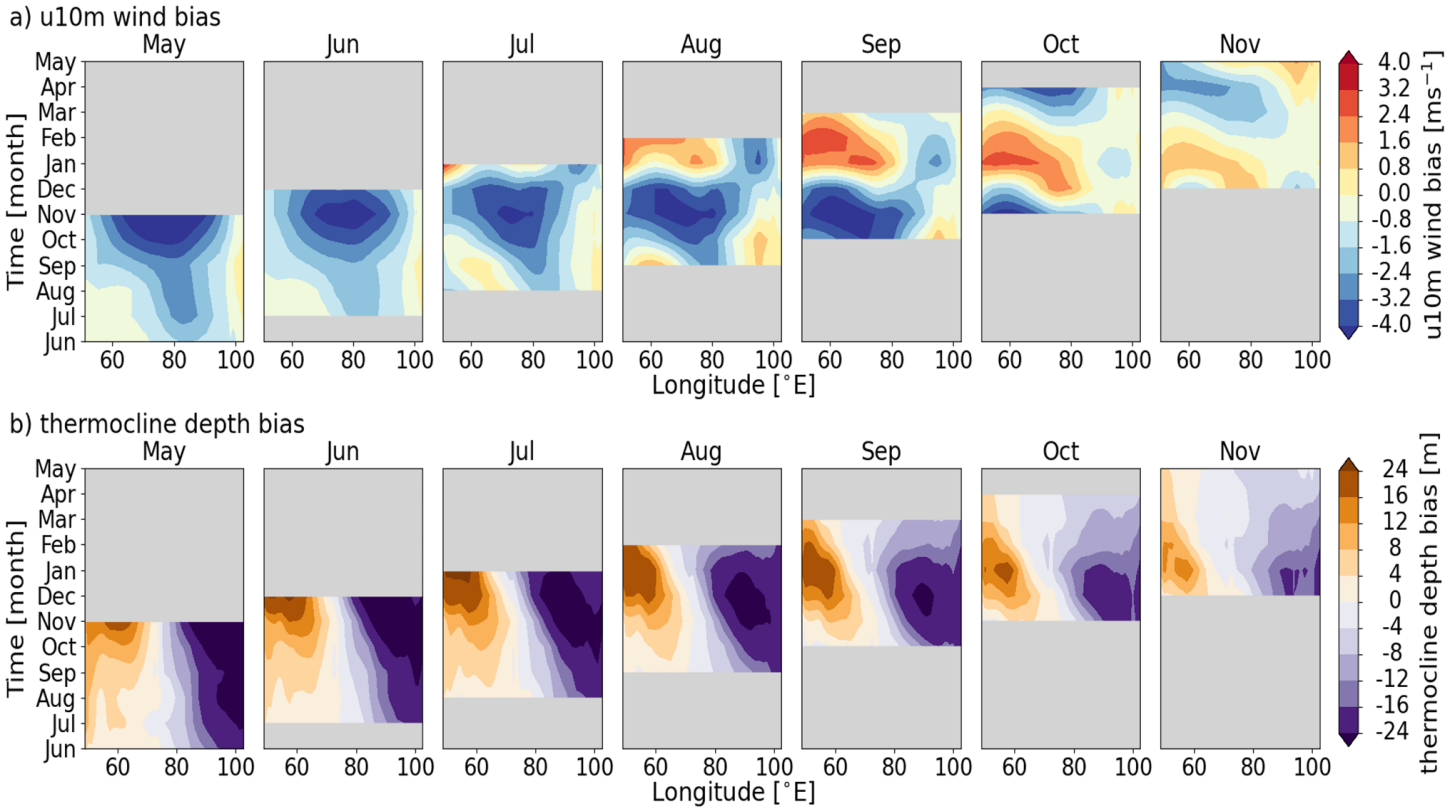
Hovmöller diagram (time versus longitude) of climatological monthly mean biases in a) 10 m zonal wind (compared against ERA5) and b) thermocline (20 °C isotherm) depth (against ORAS5), latitudinally averaged 5 ${^{\circ }}$S-5 ${^{\circ }}$N, initialised from May to November (columns) from 1993–2016. Ensemble mean of 28 ensemble members from four initialised runs (1st, 9th, 17th, 25th) per month, each with 7 ensemble members. Panel subtitles indicate the hindcast initialisation months, and time increases up the page in each case

Given the focus on the EEIO and the suspicion that the circulation bias, related to the boreal summer monsoon, plays a crucial role in driving the IOD-like SST response, the evolution of near-surface winds and thermocline depth across the basin is examined. Figure 4 shows the development of coupled mean state biases in 10 m zonal wind (u10m) and thermocline depth (using the 20 °C isotherm as a proxy) across the equatorial Indian Ocean for May-November initialisations. The range of start months, from May to November, is chosen to examine how the biases in the subsurface ocean evolve from the pre-monsoon period through to the end of the autumn season, the period across which we have shown the biases in SST and precipitation to develop most rapidly.

Hindcasts initialised from May exhibit anomalous 10 m easterly winds originating in the eastern half of the basin, and shallower thermocline depth in the EEIO from June onwards (Fig. 4a and b), which indicates a coupled feedback that leads to upwelling of deeper, cooler water to the surface, resulting in colder SSTs than observations. Johnson et al. (2017) found similar characteristics of the anomalous SST and circulation over the Indian Ocean in GloSea5, which showed that this coupled mean state bias in the IO is related to the anomalous upward tilt of the thermocline to the east compared to observations.

The easterly wind bias strengthens and extends westward after the boreal summer monsoon onset in June, reaching a maximum in boreal autumn, likely influenced by the monsoon circulation bias along the Sumatran coast (Fig. 4a). In hindcasts starting from May–September, the strengthening of erroneous easterlies in the central equatorial Indian Ocean during SON leads to the deepening of the thermocline in the west and shoaling in the east compared to observations (Fig. 4a and b), via the positive Bjerknes feedback. The coupled feedback, with an erroneous upward tilt of the thermocline toward the EEIO, relates to the large cold and dry biases there in SON. Hindcasts initialised in August-November show biases in thermocline depth reducing across the equatorial Indian Ocean from December to February of the following year.

The comparison of JJA and SON mean state biases in GloSea6 reveals a predominantly warm SST bias across the equatorial Indian Ocean, developing into a distinct dipole pattern with a warm (wet) bias in the WEIO and cold (dry) bias in the EEIO as lead time increases in JJA and SON from LM0 to LM2 (Fig. 1). Investigating the evolution of coupled biases in the WEIO and EEIO showed that the boreal summer monsoon circulation bias in the EEIO during JJA likely influences the growth of the overall dipole pattern of biases in SST, precipitation, and the subsurface ocean into SON (Figs. 2 and 4). The seasonal evolution of coupled regional biases in the equatorial Indian Ocean begins with a cold SST and dry bias in the EEIO in JJA (Fig. 2b and d), accompanied by erroneous zonal 10 m easterly winds and a shallower thermocline depth (Fig. 4a and b). This is followed by the strengthening of 850 hPa (not shown) and 10 m (Fig. 4a) easterly zonal wind biases through the JJAS months over the central equatorial Indian Ocean, and by a wet precipitation bias in the WEIO in SON (Fig. 2c). These biases reflect a positive IOD-like pattern, amplified by the Bjerknes feedback, linking SST, wind, and precipitation biases, and highlight the strong seasonal dependence of the coupled biases in the equatorial Indian Ocean.

### Representation of SST variability over the Indian Ocean and the IOD

In the previous section, JJA and SON biases in the atmosphere and subsurface ocean over the WEIO and EEIO were assessed. Here, the influence of these coupled mean state biases on the simulated interannual variability over the equatorial Indian Ocean, including IOD characteristics, is examined.

**Fig. 5:**
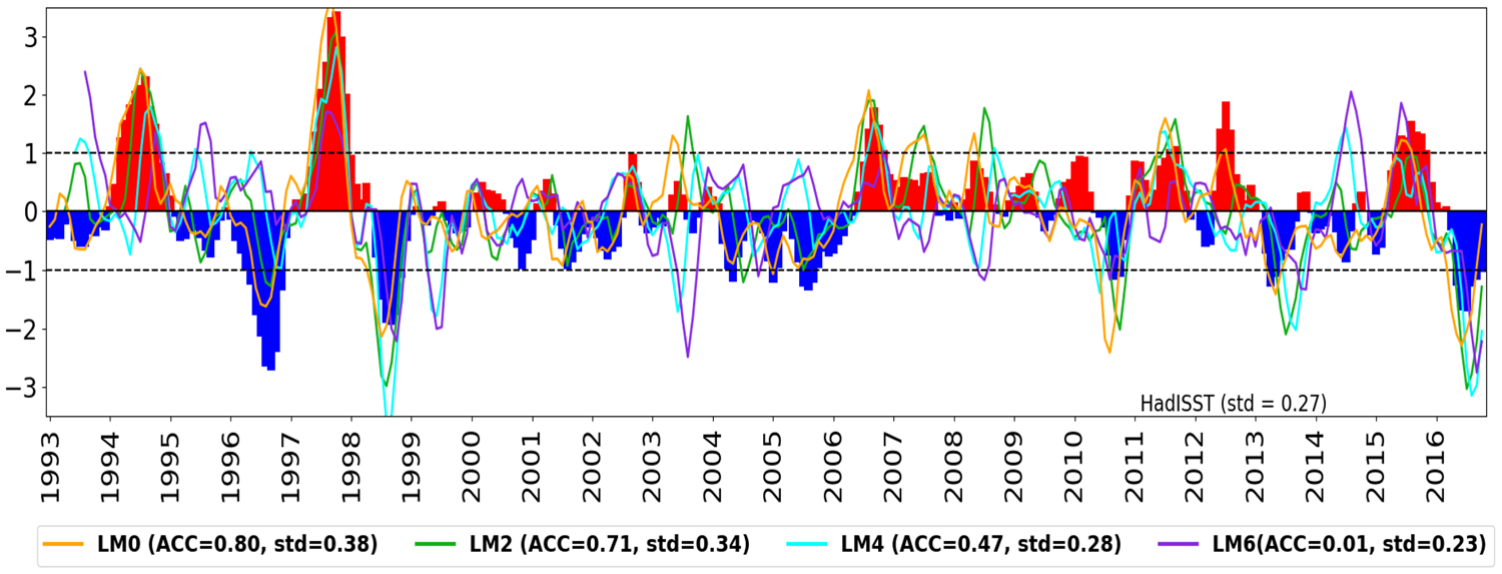
Time series of monthly DMI in HadISST (bars) and GloSea6, normalised by its standard deviation, at 0-month (orange line), 2-month (green line), 4-month (cyan line) and 6-month (purple line) lead times from 1993 to 2016. The ACC between the observed and predicted DMI is included, and the standard deviation of the predicted DMI prior to normalisation is calculated. The observed and predicted DMI have been smoothed with a 3-month running mean

Figure 5 shows the forecast DMI compared against observations for different lead times. The correlation between the observed and GloSea6 DMI at LM0 and LM2 is generally well forecast, with ACC values of 0.80 and 0.71, respectively, exceeding the commonly used ACC threshold of 0.5 (e.g., Zhao and Hendon 2009; Song et al. 2022). An ACC of 0.5 is used to indicate moderate forecast skill, which is comparable to using the climatological average as the forecast. In comparison to the ACC skill at LM0 and LM2, the forecast skill of the predicted DMI at LM4 and LM6 is relatively lower. At LM0, GloSea6 predicts stronger positive and negative IOD events compared to LM2, LM4, and LM6. For example, the magnitudes of the negative and positive IOD events observed in 1996 and 1997, respectively, are overestimated at LM0 compared with longer lead times. This is reflected in the measure of the predicted IOD amplitude, defined as the standard deviation of the GloSea6 DMI, with the highest value of 0.38 ${^{\circ }}$C at LM0. Calculating the ACC values and amplitudes for the DMI at the individual poles of the IOD reveals that the EEIO DMI has consistently lower ACC and higher IOD amplitude compared to the WEIO DMI for all lead times (LM0 to LM6) (not shown).

**Fig. 6:**
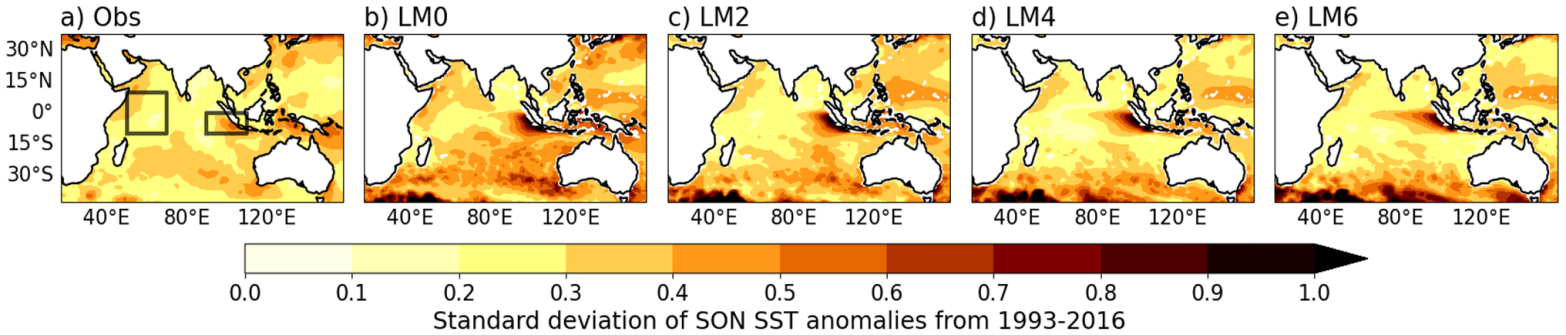
Spatial distribution of the standard deviation of SON SST anomalies, as a measure of SST variability, in **a** HadISST and **b**–**e** GloSea6 at LM0, LM2, LM4 and LM6. For each lead time, SON is obtained by averaging the monthly ensemble means for September, October, and November, each comprising 28 members

Examining the standard deviation of SST anomalies in SON, an important season during which the IOD peaks, shows a large SON SST variability over the EEIO, particularly off the coasts of Sumatra and Java (Fig. 6). This suggests that the larger IOD variability in the GloSea6 DMI compared to observations is likely due to increased SON SST variability over the EEIO. A possible hypothesis is that the larger SST variability in the EEIO in GloSea6 may be related to the erroneous easterlies in the central equatorial Indian Ocean, which strengthen and extend westward after the onset of the summer monsoon in June, peaking in SON (Fig. 4a). This hypothesis is supported by the findings of Johnson et al. (2017) who demonstrated that coupled mean-state biases in the EEIO lead to errors in representing the IOD as a mode of variability in GloSea5, thereby reducing its ability to predict the Indian monsoon circulation. Here, we have shown that the strengthening of the easterly wind bias during SON leads to a deepening of the thermocline in the west and shoaling in the east (Fig. 4), reinforcing the already shallow SON climatological thermocline of GloSea6 in the EEIO (not shown). The easterly wind bias, combined with a shallower thermocline in the EEIO, suggests that even small fluctuations in wind are likely to quickly lead to changes in upwelling. This may in turn lead to rapid adjustments in SST, as the thermocline tilt shoals in the east making the region particularly responsive to wind variations.

To examine the representation of observed positive and negative IOD events, a composite analysis of the SON hindcast ensemble mean is performed. Here, positive and negative IOD events are classified when the observed normalised DMI time series exceeds 1 standard deviation for September-November (Fig. 5). During the full hindcast period of 1993–2016, seven positive and six negative IOD events are identified in the observations.

**Fig. 7:**
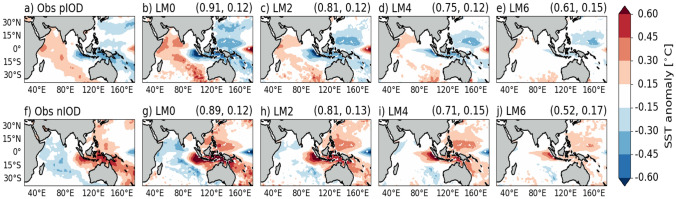
Composite maps of SON SST anomalies of positive IOD events in **a** HadISST and **b**–**e** in GloSea6 at 0 to 6-month lead times. Panels f–j) as in a-e) for composite maps of negative IOD events. PCC and RMSE [°C] are calculated between HadISST and GloSea6, and shown in parenthesis at the top right-hand corner of each panel. The positive IOD composite includes the years 1994, 1997, 2002, 2006, 2011, 2012, and 2015, while the negative IOD composite includes 1996, 1998, 2001, 2005, 2010, and 2016

At LM0, it is evident that GloSea6 exhibits larger SST anomalies over the WEIO and EEIO compared to observations for both phases of the IOD (Fig. 7b and g). For instance, the simulated positive IOD event shows colder SSTs in the EEIO and warmer SSTs in the WEIO than observed, suggesting a stronger positive IOD. This likely relates to the SON mean state biases in SST and circulation, characterised by a positive IOD-like pattern, that may be amplified during a positive IOD event. Likewise, a stronger negative IOD event relative to observations is simulated at LM0, accompanied by a dipole pattern of colder anomalies in the WEIO and much warmer SST anomalies in the EEIO than observed. Such large SST anomalies in the EEIO persist at longer lead times of up to 4 months for a positive IOD and 6 months for a negative IOD (Fig. 7d and j). Generally, the positive and negative IOD composites of SON SST anomalies at LM0 exhibit large-scale patterns in the Indian Ocean that are comparable to observations, with pattern correlations of 0.91 and 0.89, respectively (Fig. 7a, b, f and g). Figure 7 shows that the pattern correlation decreases, while the RMSE increases, with increasing lead time up to LM6 for both positive and negative IOD composites.

**Fig. 8:**
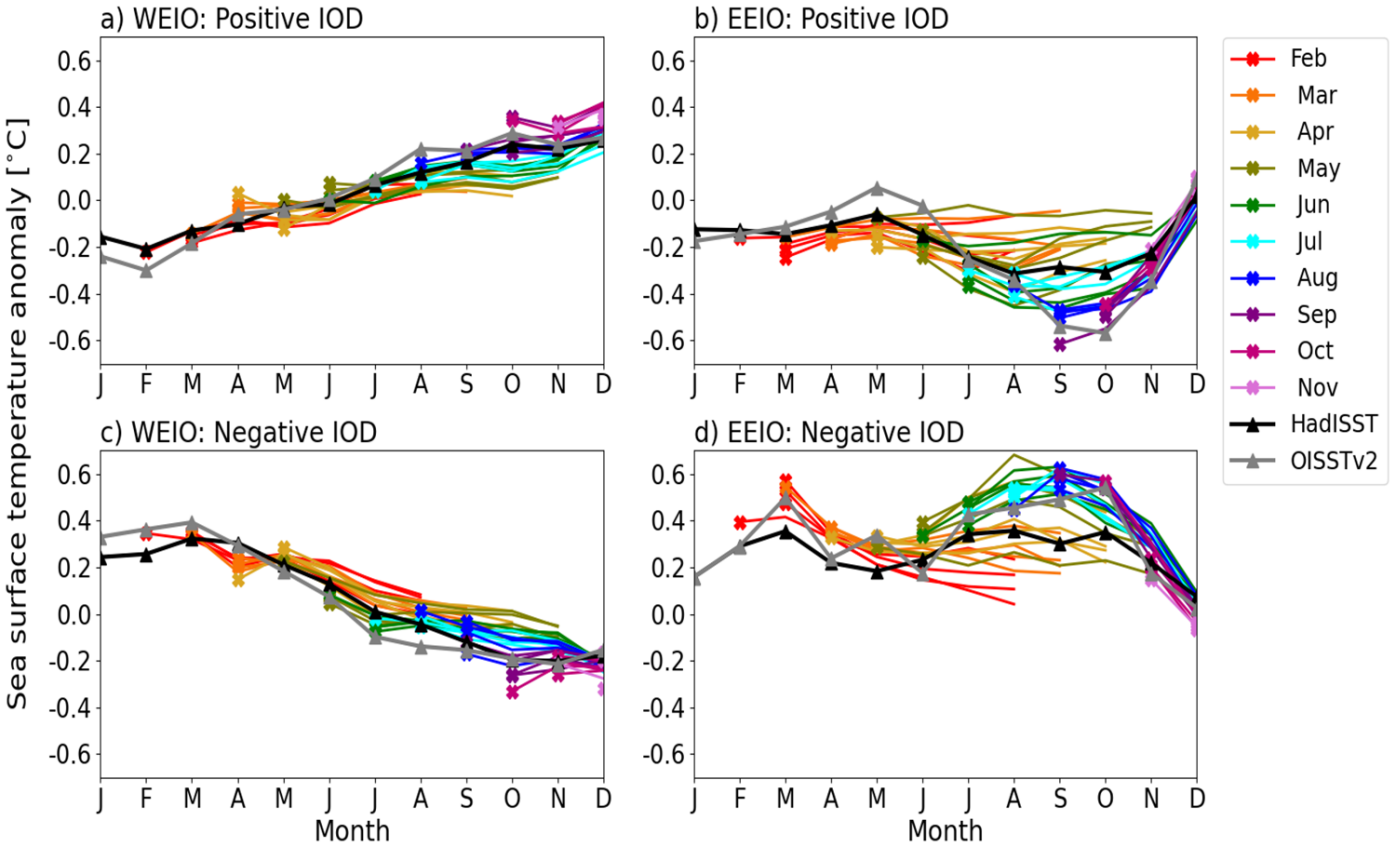
Monthly SST anomalies during positive (top) and negative (bottom) IOD events compared against HadISST (black line) and OISSTv2 (grey line) observations over the WEIO (**a**, **c**) and EEIO (**b**, **d**). Monthly anomalies are calculated by taking the difference against monthly climatological SST over the 1993–2016 hindcast period. Solid coloured lines represent the monthly ensemble means from 28 members: seven from each of four monthly start dates (1st, 9th, 17th, 25th)

Analysing the evolution of SST anomalies during SON for positive and negative IOD events reveals that these anomalies are poorly simulated in the EEIO compared to the WEIO from February to October start months. In Fig. 8, we further examine the seasonal cycle of monthly SST anomalies at both poles for positive and negative IOD events. The simulated IOD SST anomalies are compared against two observational datasets (HadISST and OISSTv2). Notably, these datasets exhibit larger observational uncertainty in the EEIO than in the WEIO, particularly during SON.

The seasonal cycle of SST anomalies over the WEIO generally match observations across different start months during the positive and negative IOD. GloSea6 is able to capture the observed warming in the WEIO during the development and mature phases of a positive IOD, specifically from June to November (Fig. 8a). Similarly, the observed cooling from June to November, associated with the evolution of SST anomalies in the WEIO during a negative IOD, is well represented (Fig. 8c).

In the EEIO, GloSea6 hindcasts started in February-May struggle to simulate the observed evolution of the cold SST anomalies, associated with a positive IOD, from June to November particularly in the SON months. These EEIO SST anomalies are underestimated and do not reach the observed cold anomalies during SON, the mature phase of the IOD (Fig. 8b). In contrast, when hindcasts are started in June-October, the simulated EEIO SST anomalies in SON during a positive IOD are generally overestimated and are much colder than those in HadISST (Fig. 8b). The colder SON EEIO SST anomalies simulated following September-November starts, compared to HadISST, (Fig. 8b) are consistent with the larger SON EEIO SST anomalies at LM0 relative to HadISST in Fig. 7b.

A similar pattern of evolution occurs with the warm SST anomalies in the EEIO during a negative IOD, where the observed warming is not well captured compared to HadISST, with colder SST anomalies in June to November for February to March starts, and warmer anomalies following June to October starts (Fig. 8d). Thus, it is evident in Fig. 8b and d, that the SST anomalies in the EEIO are poorly represented during the development and peak of the positive and negative IOD events when compared against HadISST.

**Fig. 9:**
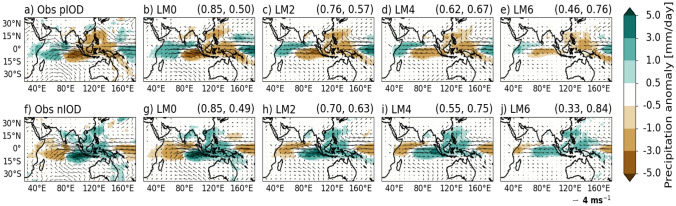
As in Fig. 7 but for SON precipitation (shaded) and wind (vectors) anomalies compared to GPCP precipitation and ERA5 winds, respectively. PCC and RMSE [mm/day] are calculated between GPCP and GloSea6 precipitation and shown in parenthesis at the top right-hand corner of panels (**b**–**e**) and **g**–**j**)

The precipitation and circulation anomalies associated with IOD SSTs for SON are shown in Fig. 9. Consistent with the stronger positive IOD and negative IOD than observed at LM0, the precipitation anomalies over the WEIO tend to extend further into the central Indian Ocean, off the equator to the north near Sri Lanka, for both positive and negative IOD events. Although the low-level circulation anomalies have considerably weakened for positive and negative IOD events, the precipitation anomalies persist in the EEIO and extend into the central equatorial Indian Ocean up to 6 months following initialisation (Fig. 9e and j). The precipitation anomalies over the EEIO at LM6 coincide with the SST anomalies over the region at the same lead time (Fig. 7e and j).

The pattern correlation of the SON precipitation anomalies compared to observations weakens as lead time increases, similar to the SST anomalies shown in Fig. 7. The dipole spatial pattern of precipitation anomalies over the IOD poles, and a large region of the Maritime Continent, shows comparable features. For instance, the magnitude and spatial distribution of precipitation over Indonesia and the Maritime Continent closely resemble observations at LM0.

Results indicate that the ability of GloSea6 to simulate observed IOD SST variability is strongest at short lead times, despite the larger monthly DMI amplitude and SON SST variability over the EEIO compared to HadISST (Figs. 5 and 6). The high ACC of the DMI at LM0 and LM2, along with pattern correlations of over 0.7 for SST and precipitation (Figs. 5, 7 and 9), suggests that GloSea6 may offer valuable potential for forecasting the IOD at short lead times. This section has shown that the large SON SST variability in the EEIO, compared to the WEIO (Fig. 6), likely relates to the poor representation of the evolution of SON SST anomaly in the EEIO during positive and negative IOD events relative to HadISST (Fig. 8).

### Predictability of the IOD

The previous section showed the ability of GloSea6 to represent positive and negative IOD phases at their maturity in SON for lead times of up to 6 months. Here, we assess the predictability of the IOD during its developing and mature phases by examining the monthly SST anomalies of the DMI as a function of lead time and different initialisation times.

**Fig. 10:**
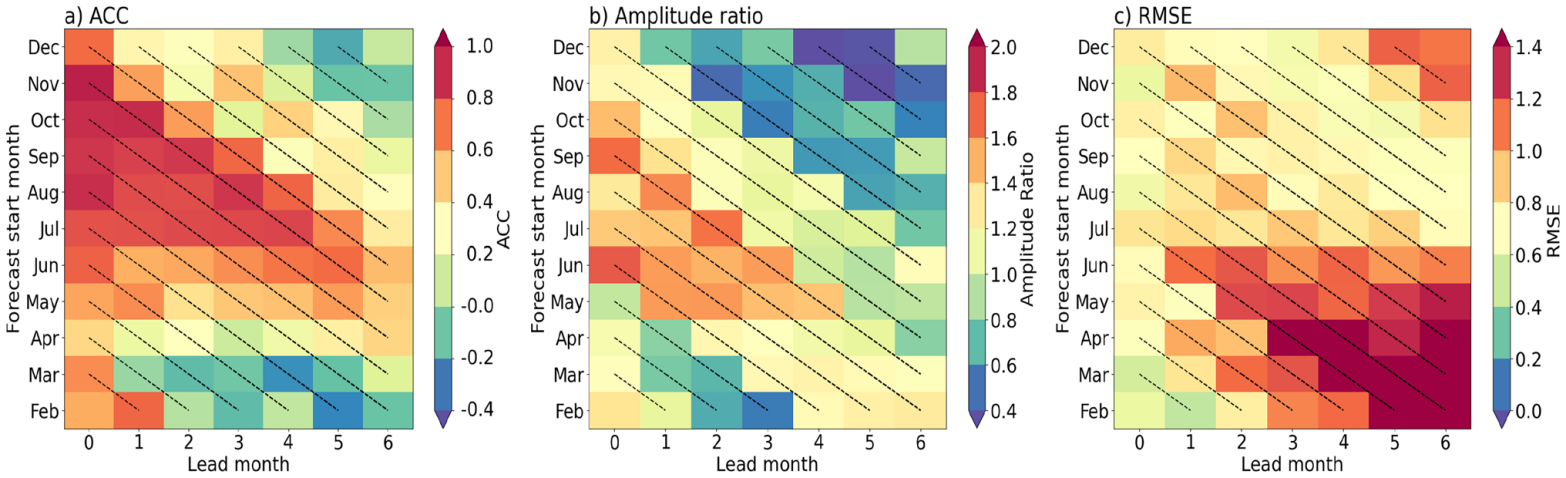
Skill metrics of the normalised monthly DMI as a function of lead month and forecast start months in **a** ACC, **b** amplitude ratio of the DMI predictions (ratio of the standard deviation of the GloSea6 DMI to that of the observed) and **c** RMSE between the predicted and observed DMI. The dashed diagonal lines indicate consistent verification months following forecast initialisation

Figure 10a demonstrates that an IOD, in its developing and mature phases, can be well predicted (defined by an ACC of 0.5 or higher) at up to 4–5-months lead time when initialised in July. In addition, GloSea6 shows good predictive skill of the IOD at up to 6 months when initialised in June, following the onset of the boreal summer monsoon. The mature phase of the IOD, which usually peaks during SON, can be predicted as early as July. The high pattern correlation between the observed and simulated composites of SON IOD SST anomalies at LM0, shown in Fig. 7, is consistent with the skillful prediction for the SON months at LM0 when initialised in September-November (Fig. 10a). Another notable feature of the prediction skill in GloSea6 is the winter predictability barrier in the decaying phase of the IOD, indicated by the rapid decline of ACC skill in boreal winter when initialised in August-November. Such a feature has been found in a fully coupled forecast system (Luo et al. 2007) regardless of the start month, and in a coupled GCM (Feng et al. 2014). Another deterministic skill metric, the IOD amplitude ratio, is shown in Fig. 10b. As discussed in the previous section, GloSea6 simulates IOD events with amplitudes that are high compared to observations. Here, the amplitude ratio is determined as the ratio of monthwise standard deviation of the predicted monthly DMI to that of the observed standard deviation. Thus, an amplitude ratio of 1 indicates a perfect match between GloSea6 and observations. Stronger than observed amplitude of the predicted IOD, with ratios greater than 1, is simulated when started in June-September with up to 2 months lead. Similar to the ACC skill, the amplitude ratio falls rapidly in boreal winter for hindcasts initialised in August-December. Although GloSea6 predicts strong IOD events in SON, the RMSE scores show low prediction errors, less than 0.5, when started in September-November (Fig. 10c). The highest prediction errors of greater than 0.6 tend to be simulated for hindcasts started in February-May, which may be attributed to the large mean-state bias in SST that grows into SON over the EEIO following initialisation in spring shown in Figure 2b. An examination of ACC and RMSE skill scores of the separate poles of the IOD reveals that the EEIO DMI has lower ACC and higher RMSE values than the WEIO DMI for up 4 months lead time when initialised in July-September (not shown).

Overall, while GloSea6 demonstrates strong prediction skill for IOD events, especially when initialised in late boreal summer or early autumn, it shows limitations in the boreal winter months. Specifically, GloSea6 demonstrates skillful prediction of the IOD during its developing and mature phases when initialised in July. In addition, GloSea6 tends to predict stronger IOD events than observed, with amplitude ratios higher than 1 for forecasts started between June and October. However, prediction errors are higher for forecasts initialised in March-June.

## Conclusion

Despite the significance of the WEIO and EEIO as key regions of IOD SST variability, few studies have specifically explored the coupled mean-state biases in these regions and linked their impacts to IOD predictability (Zhao and Hendon 2009; Shi et al. 2012). The presence of these regional biases and their role in modulating local climate and weather patterns over countries surrounding the Indian Ocean through IOD atmospheric teleconnections highlights the importance of accurately representing the underlying coupled processes in both the mean climate and variability in the WEIO and EEIO. Most recent research has focused on mean-state biases and their sources across the broader equatorial Indian Ocean region, such as the persistent positive IOD-like bias in SST, precipitation, and circulation which is well-documented in coupled GCMs (Li et al. 2015; Long et al. 2020). In comparison, the evolution and interannual variability of coupled biases in the WEIO and EEIO remain less studied. This study, therefore, focused on these regional biases, examining their evolution on seasonal and interannual timescales and linking them to IOD SST variability and prediction. The analysis of coupled initialised GloSea6 seasonal hindcasts aimed to answer the questions presented at the start of the study.

**a)** *How do mean-state biases in the atmosphere and subsurface ocean evolve in the WEIO and EEIO?* The analysis focused on the evolution of coupled mean state biases in JJA and SON, given their importance for IOD development and maturity, respectively. Both JJA and SON mean state biases in SST, precipitation, and 850 hPa winds at LM0 (0-month lead time) showed a predominantly warm SST bias across the equatorial Indian Ocean, along with significant cold and southeasterly wind biases over the EEIO. This cold bias in the EEIO intensifies by LM2 (2-month lead time), forming a distinct dipole pattern with warming in the WEIO and cooling in the EEIO. At LM2, the related JJA and SON precipitation biases show a consistent dipole pattern, resembling a positive IOD with a wet bias in the WEIO and a dry bias in the EEIO.

Investigation of the seasonal cycles of SST and precipitation over the WEIO and EEIO revealed a persistent WEIO warm bias throughout the year, in contrast to a EEIO cold bias that gradually increases in magnitude from JJA to SON. Correspondingly, an EEIO dry precipitation bias rapidly develops in JJA and SON, which contrasts the WEIO wet precipitation bias that only peaks later in SON. Analysis of the seasonal evolution of the biases in the atmosphere and subsurface ocean showed that the EEIO plays the leading role in the development of the large SST and precipitation biases in SON, especially for forecasts initialised in May. The sequence begins with a circulation bias in the EEIO during JJA, characterised by erroneous easterlies and a shallow thermocline, likely related to the boreal summer monsoon circulation. These biases in the wind and thermocline lead to upwelling of cooler subsurface water, reinforcing the cold SST bias and dry conditions in the EEIO in JJA. At the same time, the 850 hPa and 10 m easterly wind biases in the central equatorial Indian Ocean strengthen through JJAS, amplifying into SON via the Bjerknes feedback. This, in turn, leads to the intensification of the WEIO wet bias by SON. This seasonal sequence, beginning with the monsoon-driven circulation bias in JJA in the EEIO and culminating in a large wet bias in the WEIO in SON, highlights the seasonal dependence of coupled biases in these regions and the leading role of the EEIO in initiating coupled feedbacks across the equatorial Indian Ocean. Notably, Karrevula et al. (2024) demonstrated using the North American Multi-Model Ensemble models that forecasts initialised in May capture warming in the central Indian Ocean due to strengthened equatorial easterlies, which they identified as critical in modulating the frequency of extreme positive IOD events and their impact on summer monsoon precipitation from June to November

**b)** *What influence do the WEIO and EEIO regional biases have on the representation and predictability of the IOD?*

Results show that the GloSea6 DMI time series of monthly SST anomalies has a high anomaly correlation coefficient compared to the HadISST DMI at short lead times (LM0 and LM2). The high ACC skill of the predicted monthly DMI at LM0 is consistent with the high pattern correlation of over 0.8 between the observed and simulated composites of SON SST and precipitation anomalies in both positive and negative IOD events. However, results also showed that the amplitude of monthly DMI is larger compared to HadISST from LM0 to LM4 (0–4 month lead times), indicating higher IOD SST variability in GloSea6. Additionally, examining the separate poles of the IOD reveals lower ACC and higher IOD amplitude for the EEIO than the WEIO DMI for all lead times (0–6 month lead times). Investigating the SST variability in SON, during which the IOD peaks, showed a larger SON SST variability in the EEIO compared to HadISST. A possible hypothesis is that the erroneous easterlies and shallow thermocline depth in the EEIO make the region highly sensitive to small wind fluctuations, which can rapidly alter upwelling and SST. This aligns with the findings of Johnson et al. (2017), who showed that coupled mean-state biases in the EEIO lead to errors in representing the IOD as a mode of variability in GloSea5. The analysis of the seasonal cycle of SST anomalies over the WEIO and EEIO during positive and negative IOD events showed a difference in how well GloSea6 captures the observed SST anomalies in each region. In the EEIO, cold SST anomalies in SON are overestimated relative to HadISST, especially when initialised from June onwards. However, in the WEIO, GloSea6 closely matches the observed evolution of warm SST anomalies into SON during the mature phase of a positive IOD, regardless of initialisation dates.

Assessing the predictability of GloSea6 showed considerable skill in forecasting the IOD during its developing and mature phases, especially when initialised in June and July. The model demonstrates skillful prediction of IOD SST anomalies in SON, achieving an ACC of 0.5 or higher for forecasts started as early as July. Notably, the highest predictive skill for the IOD occurs when initialised between September and November, coinciding with the peak of observed IOD events. Although GloSea6 shows reasonable predictive skill for the IOD, it encounters a significant winter predictability barrier, resulting in a rapid decline in skill during the IOD’s decaying phase. This limitation has also been found in another fully coupled forecast system (Luo et al. 2007), regardless of the start month, and in a coupled GCM (Feng et al. 2014). GloSea6 has also been shown to overestimate the intensity of IOD events, particularly during the development phase in JJA, as indicated by amplitude ratios exceeding 1 when comparing the predicted DMI to the observed DMI. Additionally, RMSE scores of the GloSea6 DMI, calculated against HadISST, reveal large prediction errors for SON when initialised in June. This suggests that the monsoon circulation in JJA likely plays an important role in shaping the mean state and variability in the equatorial Indian Ocean.

These results suggest that reducing regional coupled biases over the equatorial Indian Ocean, particularly in the EEIO, could lead to improved IOD forecasts during SON in GloSea6, potentially from as early as May. Our analysis highlights the strong influence of atmospheric circulation biases during and after the onset of the summer monsoon in driving surface cooling through wind-driven upwelling, particularly over the EEIO.

Further research could perform ’nudging’ sensitivity experiments in the EEIO, such as the technique implemented by Crétat et al. (2017) and Martin et al. (2021), to disentangle the local and remote contributions of the oceanic and atmospheric components to the coupled processes in the Indian Ocean. Martin et al. (2021) applied atmospheric nudging by relaxing the winds and air temperature back to reanalysis at all model levels over the whole globe and chosen sub-domain regions that may be local and remote sources of Indian Ocean systematic biases in the model.

While this present study focused on regional processes within the Indian Ocean, additional sources of bias may arise from remote influences. For example, recent studies have highlighted the potential role of the Southern Ocean in IOD variability and predictability (e.g. Zhang et al. 2020; Feba et al. 2021). Zhang et al. (2020) propose a mechanism in which cold SST anomalies and anomalous subtropical high pressure in the southern Indian Ocean generate southeasterly winds that strengthen the monsoon off the coast of Sumatra during May-August, independent of ENSO. The enhanced southeasterly winds induce early SST cooling via upwelling and latent heat loss, initiating an early IOD onset over the eastern IOD pole. This mechanism highlights the importance of the summer monsoon atmospheric circulation over the EEIO as a critical region in driving coupled processes that can influence the Indian Ocean mean state and variability.

In addition, we recognise the potential role of the equatorial Pacific Ocean and the representation of the Indonesian Throughflow that may influence the coupled biases in the Indian Ocean and IOD simulation in GloSea6. Annamalai et al. (2003) suggest that equatorial Pacific SST anomalies can modulate EEIO conditions through changes in the Walker circulation during boreal spring, potentially triggering IOD events. More recently, McKenna et al. (2020) found that coupled GCMs with warmer SSTs in the western Pacific tend to exhibit stronger IOD events. Further research is needed to explore these broader Indo-Pacific interactions that can influence IOD-like mean state biases and potentially impact IOD prediction in forecasts systems.

Overall, this study highlights of addressing regional biases in the WEIO and EEIO is essential for improving IOD representation in coupled forecast systems like GloSea6 to enhance the predictability of climate impacts over the countries surrounding the Indian Ocean.

## Data Availability

Model data used in this study are available to research collaborators upon request.

## Code availability

Due to intellectual property rights restrictions, we cannot provide either the source code or the documentation papers for the Met Office Unified Model.
